# Smoothness of Gait in Healthy and Cognitively Impaired Individuals: A Study on Italian Elderly Using Wearable Inertial Sensor

**DOI:** 10.3390/s20123577

**Published:** 2020-06-24

**Authors:** Massimiliano Pau, Ilaria Mulas, Valeria Putzu, Gesuina Asoni, Daniela Viale, Irene Mameli, Bruno Leban, Gilles Allali

**Affiliations:** 1 Department of Mechanical, Chemical and Materials Engineering, Piazza d’Armi, 09123 Cagliari, Italy; imulas89@gmail.com (I.M.); bruno.leban@dimcm.unica.it (B.L.); 2 Center for Cognitive Disorders and Dementia, Geriatric Unit SS. Trinità Hospital, Via Romagna 16, 09127 Cagliari, Italy; valeria.putzu@atssardegna.it (V.P.); gesuina.asoni@atssardegna.it (G.A.); danielaviale@tiscali.it (D.V.); dott.irenemameli@gmail.com (I.M.); 3 Department of Clinical Neurosciences, Division of Neurology, Geneva University Hospitals and Faculty of Medicine, University of Geneva, 1205 Geneva, Switzerland; Gilles.Allali@hcuge.ch; 4 Department of Neurology, Division of Cognitive & Motor Aging, Albert Einstein College of Medicine, Yeshiva University, Bronx, NY 10461, USA

**Keywords:** gait, smoothness, older adults, accelerometer, inertial measurement unit (IMU)

## Abstract

The main purpose of the present study was to compare the smoothness of gait in older adults with and without cognitive impairments, using the harmonic ratio (HR), a metric derived from trunk accelerations. Ninety older adults aged over 65 (age: 78.9 ± 4.8 years; 62% female) underwent instrumental gait analysis, performed using a wearable inertial sensor and cognitive assessment with the Mini Mental State Examination (MMSE) and Addenbrooke’s Cognitive Examination Revised (ACE-R). They were stratified into three groups based on their MMSE performance: healthy controls (HC), early and advanced cognitive decline (ECD, ACD). The spatio-temporal and smoothness of gait parameters, the latter expressed through HR in anteroposterior (AP), vertical (V) and mediolateral (ML) directions, were derived from trunk acceleration data. The existence of a relationship between gait parameters and degree of cognitive impairment was also explored. The results show that individuals with ECD and ACD exhibited significantly slower speed and shorter stride length, as well as reduced values of HR in the AP and V directions compared to HC, while no significant differences were found between ECD and ACD in any of the investigated parameters. Gait speed, stride length and HR in all directions were found to be moderately correlated with both MMSE and ACE-R scores. Such findings suggest that, in addition to the known changes in gait speed and stride length, important reductions in smoothness of gait are likely to occur in older adults, owing to early/prodromal stages of cognitive impairment. Given the peculiar nature of these metrics, which refers to overall body stability during gait, the calculation of HR may result in being useful in improving the characterization of gait patterns in older adults with cognitive impairments.

## 1. Introduction

Optimal locomotion capabilities represent a critical element in ensuring successful aging. Mobility is not only an important co-factor that influences life expectancy [1,2], but also plays a relevant role in the self-perception of aging [3], social participation [4], independence and overall quality of life [5].

The physiologic decline in quality and the effectiveness of sensory, vestibular and proprioceptive inputs, associated with the loss of muscle strength [6,7,8], alter several main features of gait pattern. Elderly individuals present reduced gait speed, stride length and cadence, as well as increased stance and double support phase duration [9]. Taken together, these features indicate the adoption of cautious gait, a strategy necessary to counteract the loss of stability and, thus, reduce the risk of falls [10].

Although gait has long been considered mostly an automatic task, in the last decades, it has been postulated that cognitive performances (mainly executive functions) provide an essential contribution, especially through the regulation of postural control (strongly implicated during walking), owing to their role in the management of axial musculature and in the integration of visual, vestibular, proprioceptive and sensory feedback. The sum of required cognitive resources becomes even more relevant when environmental conditions tend to reduce the automaticity of the task, as occurs in the case of uneven terrain and in the presence of concurrent motor/cognitive tasks (i.e., dual-task). Instability thus increases and overall gait performance may be compromised as a result [11]. It has also been observed that early disturbances in cognitive processes, such as attention, executive functions and working memory, often coexist with slower gait speed, increased stride time variability and greater instability [12,13,14].

Owing to its essentiality for most activities of daily living and considering that a walking test can easily be performed even by an individual with severe cognitive impairment, gait is probably the most thoroughly investigated motor task in describing the impact of cognitive performance on overall mobility. While basic information on speed can be obtained from simple timed tests carried out using a stopwatch (like the 10-m walking test), fine details on the kinematics and kinetics of gait require more complex equipment, such as motion capture systems, force platforms and surface electromyography. In this scenario, for more than a decade, interest in the possibility of employing accelerometers and inertial measurement units (IMUs i.e., devices composed of tri-axial accelerometer, gyroscope and magnetometer) in human movement analysis has been increasing [15]. To date, low-cost, wearable and miniaturized IMUs featuring high reliability and easiness of use are available. Their performance is increasingly close to those of more expensive and complex equipment. Such devices have been successfully employed to perform several tests on balance, gait and functional mobility under ecological conditions in older adults, with and without cognitive impairments [16,17,18,19,20,21]. Particularly attractive for daily clinical routines is the use of a simple setup consisting of a single unit [22], since the analysis can be performed by a non-specialized person (e.g., nurse, physical therapist, physician) in a clinical/ambulatory setting, and under very ecologic conditions in a relatively short time.

A gait analysis assisted by IMUs can provide a large set of parameters, which includes the main spatio-temporal parameters (i.e., speed, cadence, step/stride length and duration of stance, swing and double support phases), as well as indicators of variability, regularity and symmetry (see the review by Jarchi et al. [23] for details). In particular, the specific processing of trunk accelerations allows the extraction of less conventional metrics which, in some cases, are able to reveal subtle changes in gait that might occur, well before they become detectable in terms of conventional spatio-temporal parameters. Among them, great interest has been raised by the so-called “smoothness” of gait [24] (also defined as “step-to-step” symmetry [25]). Such a feature, quantitatively identified by a parameter called harmonic ratio (HR), provides information about overall body movement during gait, in particular with regards to its stability [26], which is different from the typical spatio-temporal parameters, which are rather focused on lower-limb movement at the distal level. The study of HR has aroused significant interest among researchers of human movement, as it allows the detecting of gait alterations in individuals with neurologic and orthopedic conditions and characterizes the changes associated with aging [27,28,29]. 

### Use of Accelerometers and IMU to Analyze Gait in Elderly with and without Cognitive Impairment

Accelerometers (alone or as part of IMU) have been used for almost three decades to investigate a wide range of aspects correlated with mobility and posture in older adults. In particular, early applications were focused on the analysis of trunk accelerations during gait, to obtain information on stability and smoothness [30,31], but with the advancement of the hardware’s technology, as well as with the refinement of the signal processing techniques, even other movement features were explored. To date, gait analysis represents the most widespread example of application for such class of devices. In the elderly population, gait analysis is typically employed to assess spatio-temporal parameters which are useful to estimate, for example, the risk of falls, or to assess the extent of functional limitations associated with orthopedic and neurologic conditions [20]. Given the simplicity of use and the fact that no special preparation of the individual to test is needed, IMU are gaining increasing popularity in the clinical testing of elderly with mild cognitive impairments, Alzheimer’s disease, or other types of dementia [17,21]. As a result, these studies allowed one to detect the existence of peculiar gait alterations (i.e., reduction of walking speed and stride length, increased variability and asymmetry, etc.), which reflect the modifications in brain structure and functions associated with cognitive deficit [17,18,21,32]. Moreover, gait data obtained from wearable accelerometers were able to discriminate different subtypes of dementia [21], thus suggesting that such devices might represent a useful tool for supporting the clinical diagnosis. 

Several studies also attempted to correlate trunk accelerations features, acquired during walking tests, with clinical characteristics of older adults with cognitive impairments. Their main findings can be summarized as follows: in comparison with unaffected individuals, older adults with cognitive impairments exhibit significant reduced value of the root mean square (RMS) and structure variability of the medio-lateral trunk acceleration [33], significant association of trunk stability measures with the white matter lesions [34] and with cognitive performance [35]. In particular, the study of Ijmker and Lamoth [35] showed that the presence of a cognitive impairment is accompanied by a decrease in smoothness of gait along the walking direction (anteroposterior, AP), as indicated by the significantly reduced value of the corresponding HR. Moreover, HR AP was found to be significantly correlated with cognitive status, as expressed by the Mini Mental State Examination (MMSE) score. However, although innovative and interesting, such findings require further verification and extension, firstly owing to the limited size of the tested sample, as well as its unbalanced composition in terms of the men to women ratio (75 to 85% of the tested individuals were men). It is also noticeable that the role of HR in the ML direction has not been clarified, being found to increase in cognitively impaired individuals, contrary to expectations. Finally, HR in the V direction was not even considered.

Based on the aforementioned considerations, the main purpose of the present study was to analyze the spatio-temporal and smoothness of gait parameters for a cohort of older adults, with and without cognitive impairments. The main hypothesis to verify was if individuals with impaired cognitive performance are characterized by altered gait patterns and reduced smoothness of gait. As a secondary goal, the existence of possible relationships between the degree of cognitive impairment and the gait parameters investigated will also be explored.

## 2. Materials and Methods

### 2.1. Participants

In the period January 2020–February 2020, 90 elderly adults aged over 65, consecutively examined at the Center for Cognitive Disorders and Dementia (in collaboration with the Geriatric Unit, “SS. Trinità” General Hospital, Cagliari, Italy), were recruited for the study. All participants were free from other neurologic conditions (e.g., Parkinson’s disease, multiple sclerosis and stroke), excluding cognitive decline. They were also free from orthopedic conditions able to interfere in mobility, and could walk independently without the need of any support, such as canes, walking frames, crutches etc. After a detailed explanation of the purposes and methodology of the study, they (or their family members/caregivers when necessary) signed an informed consent form. The study was conducted in accordance with the ethical standards of the institutional research committee, and with the 1964 Helsinki declaration and its later amendments. 

### 2.2. Neuropsychologic Assessment

After an overall clinical and geriatric assessment, participants underwent a screening of their cognitive status carried out by means of: (1) the Italian version of the Mini Mental State Examination (MMSE, [36,37]) and (2) Addenbrooke’s Cognitive Examination Revised (ACE-R, [38,39]). ACE-R is articulated across five cognitive domains, namely attention and orientation, memory, verbal fluency (related to cognitive abilities of executive function), visuospatial, and language. The overall ACE-R score ranges from 0 to 100, lower scores being indicative of greater cognitive impairment. We decided to employ both tests, because although MMSE is probably the most widespread rapid cognitive screening instrument and, as such, has a large amount of reference data available, it also suffers from several drawbacks which are partly overcome by ACE-R. 

Participants were stratified into 3 groups, according to their MMSE score, based on the cut-offs proposed by Isella et al. [40], as follows:

- Healthy controls (HC): MMSE score ≥ 24 (n = 34)
- Early cognitive decline (ECD): 18 ≤ MMSE score < 24 (n = 37);
- Advanced cognitive decline (ACD): MMSE score < 18 (n = 19);

Their anthropometric and clinical features are reported in Table 1.

### 2.3. Instrumental Gait Analysis

Gait patterns were investigated based on trunk accelerations collected using a miniaturized wearable inertial sensor (G-Sensor^®^, BTS Bioengineering, Italy), previously employed in studies involving the elderly [41,42]. The sensor was attached to participants’ lower back, at approximately the S1 vertebrae level, using a dedicated semi-elastic belt. After a brief familiarization phase, participants were requested to walk along a 30-m hallway following a straight trajectory at a self-selected speed, and in the most natural manner. During the trial, the sensor acquired, at 100 Hz frequency, the accelerations along three orthogonal axes, namely: antero-posterior (AP) corresponding to the walking direction, medio-lateral (ML), and supero-inferior (V). In order to reduce the error possibly introduced by the initial misalignment of the sensor (particularly with regards to the V direction), the participants were asked to stand still for 10 s before starting the walking trial, and the local reference system of the device was rotated in such a way as to align its vertical axis with the gravity vector [43]. Acquired data were sent in real-time via Bluetooth to a Personal Computer, where they were subsequently processed with a custom Matlab^®^ routine to calculate:

- spatio-temporal parameters of gait (namely gait speed, stride length, cadence, duration of stance, swing and double support phase expressed as a percentage of the gait cycle). The identification of the gait cycle and the subsequent extraction of such parameters was carried out by means of a peak-detection algorithm, according to the procedure described by Zijlstra [44];
- HRs for AP, ML and V directions.

The calculation of the HRs was carried out according to the procedure proposed by Menz, Lord and Fitzpatrick [31]. In short, the raw accelerometric signal is processed in the frequency domain using a finite Fourier series, and the HRs for the AP and V directions (see Equation (1)) are calculated as the ratio between the sum of the amplitudes (A) of the first ten even harmonics (which are representative of the in-phase components of the signal) and the sum of the amplitudes of the first ten odd harmonics (associated with the out-of-phase components), the latter being minimized as gait symmetry improves. Instead, the HR in the ML direction (see Equation (2)) is obtained by dividing the sum of the amplitudes of the odd harmonics by the sum of the amplitudes of the even harmonics, since the acceleration pattern exhibits one peak per stride, thus resulting in the dominance of the first harmonic and subsequent odd harmonics.

(1) $$HR_{AP-V}=\frac{\sum^{}A_{even\;harmonics}}{\sum^{}A_{odd\;harmonics}}$$

(2) $$HR_{ML}=\frac{\sum^{}A_{odd\;harmonics}}{\sum^{}A_{even\;harmonics}}$$

The interpretation of the HR values is quite straightforward, as lower values indicate a less smooth/symmetrical gait. Reference values for healthy older adults lie in the range 3–4 (for AP and V directions) and 2.1–2.6 for the ML direction [26,45,46,47,48].

### 2.4. Statistical Analysis

The existence of possible differences introduced in spatio-temporal parameters and HRs by participants’ cognitive status was assessed using a one-way multivariate analysis of variance (MANOVA) and a one-way multivariate analysis of covariance (MANCOVA), respectively. In the latter case, gait speed was included in the analysis as a covariate, given its influence on HR values [46]. The independent variable was the participant’s status (e.g., HC, ECD or ACD) and the dependent variables were the 6 spatio-temporal parameters and the 3 HRs. In both cases, the level of significance was set at *p* = 0.05, and the effect sizes were assessed using the eta-squared (η^2^) coefficient. Univariate ANOVA was carried out as a post-hoc test, by reducing the level of significance to *p* = 0.008 (0.05/6) for spatio-temporal parameters and *p* = 0.016 (0.05/3) for HRs, after a Bonferroni correction for multiple comparisons. The relationship between spatio-temporal gait parameters and cognitive status (as indicated by both MMSE and ACE-R scores) was explored using Spearman’s rank correlation coefficient rho, by setting the level of significance at *p* < 0.05. Rho values of 0.1, 0.3, and 0.5 were assumed to be representative of small, moderate, and large correlations respectively, according to Cohen’s guidelines [49]. In the case of HR, we used partial correlation coefficients, checking for gait speed. All analyses were carried out using the IBM SPSS Statistics v.23 software (IBM, Armonk, NY, USA).

## 3. Results

### 3.1. Spatio-Temporal Parameters of Gait and Harmonic Ratio

The results of the experimental test are summarized in Table 2 (comparison of the spatio-temporal and HR values across the three groups) and in Table 3 (correlation analysis between gait parameters and MMSE/ACE-R scores).

MANOVA detected a significant main effect of group on the spatio-temporal parameters of gait [F(12, 164) = 2.17, *p* = 0.016, Wilks λ = 0.74, η^2^ = 0.14 ], but the post-hoc analysis revealed that only gait speed and stride length actually differed across the tested groups. In particular, individuals with both ECD and ACD exhibited a significant reduced gait speed (0.68 and 0.63 m/s respectively vs. 0.92 m/s of HC, *p* = 0.001 in both cases) and stride length (0.81 and 0.73 m vs. 1.03 m of HC, *p* < 0.01 in both cases) with respect to unaffected participants. 

**Figure 1:**
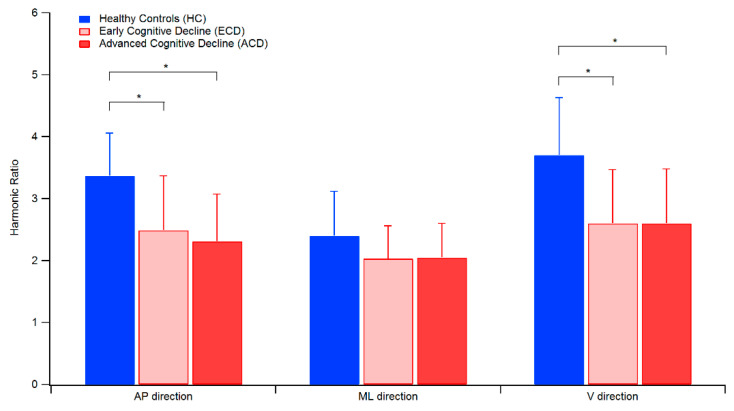
Trend of HR values for the three groups of tested elderly. The symbol * denotes a statistically significant difference after Bonferroni correction (*p* < 0.016)

Trends of the HR, calculated using the two methods previously described, are reported in Figure 1.

After controlling for gait speed, MANCOVA detected a significant main effect of individuals’ status on HR values [F(6168) = 3.42, *p* = 0.003, Wilks λ = 0.79, η^2^ = 0.11], and the post-hoc analysis revealed that HR in the AP and V directions differed significantly across the tested groups. For both directions in particular, individuals of the ECD and ACD groups exhibited HR values that were significantly lower that healthy controls, while no differences were found between the two groups of cognitively impaired elderly.

### 3.2. Correlation between Gait Parameters and Cognitive Impairment

Gait speed and stride length were positively correlated with both measures of cognitive status with coefficients similar in magnitude, while no correlations were found with the remaining gait parameters. When we checked speed on the relationship between MMSE scores and HRs, we found a significant positive partial correlation, with rho ranging from 0.21 (ML direction) to 0.32 (AP direction). Similar results were obtained in the case of ACE-R, where the coefficients varied between 0.21 (HR V direction) and 0.30 (HR in AP direction).

## 4. Discussion

### 4.1. General Considerations

The aim of this study was to quantitatively investigate the alterations of gait patterns consequent to the presence of a cognitive impairment of different severity, using a wearable inertial sensor in a clinical setting, and to explore the existence of possible relationships between gait parameters and the degree of impairment. To this end, we employed the typical spatio-temporal parameters of gait, and trunk acceleration-based measures such as HR, which provide a different point of the view of gait alterations associated with overall body stability. In particular, we attempted to extend the previous limited findings by calculating HR for all three directions (AP, ML and V), enlarging the tested sample and analyzing the correlations of HR with two different measures of cognitive performance, namely MMSE and ACE-R.

At first, consistent with most existing studies, our data confirm that the existence of cognitive impairment, even mild, is associated with significant reductions in gait speed and stride length, while cadence and phase subdivision of the gait cycle appear to be less altered. The speed reductions of individuals with cognitive impairments with respect to unaffected controls is clinically meaningful and, particularly in the case of ECD, in very good agreement with the values recently reported by Peel et al. [50], in a meta-analysis, summarizing the results of 36 studies, involving more than 29,000 participants. Participants with more advanced impairment showed slower speed (−8%) and shorter stride length (−11%) with respect to individuals with ECD, but such a change was not found to be significant and, as such, should rather be considered as a trend. Taken together, the reduction in gait speed and stride length indicate that cognitive decline influences gait strategy, through the adoption of a cautious approach that probably reflects the diminished efficiency of sensory and motor systems and attempts to achieve a more stable locomotion to reduce the risk of falls [51].

### 4.2. Smoothness of Gait

Firstly, it is to be noted that the HR values calculated in the present study for our reference group of healthy older adults are consistent, even from a quantitative point of view, with those reported in previous studies involving individuals of the same age range [45,46,47,48]. This demonstrates that, despite the variability in terms of equipment and measurement protocols, the approach based on HR analysis is reliable and robust. As regards the values observed in individuals with cognitive impairment, even after checking for gait speed (which is known to have a direct influence on HR) our data show a substantial significant decrease in smoothness for all three directions considered, although only in the case of the AP and V directions were the variations statistically significant.

In the last two decades, several studies have employed HR to investigate gait performance in older adults for different purposes, such as characterizing the changes associated with either aging [27,45] or the presence of neurological diseases [26], assessing the risk of falls [41,44] and verifying the differences between overground and treadmill walking [43]. In short, their main findings indicate that older adults feature lower HR values with respect to young individuals. Moreover, further reductions have been observed in those suffering either from recurrent falls or in the presence of neurologic conditions known to affect balance and stability, such as Parkinson’s disease [26], stroke [28] and multiple sclerosis [29]. However, only in the study by Ijmker and Lamoth [35] was the analysis of smoothness of gait applied to a small cohort of individuals with cognitive impairments, and it thus represents the only term of comparison for the findings derived by the present study. Consistently with our results, they observed a significant lower value for HR in the AP direction in cognitively impaired individuals, with respect to the unaffected elderly. In contrast, individuals with dementia exhibited higher values of HR ML with respect to both unaffected elderly and young subjects. This is contrary to the findings of our study, as participants in the ECD and ACD groups had significantly lower HR ML values than unaffected controls. The findings by Imjker and Lamoth [35] are actually quite surprising, as higher HR values indicate better smoothness of gait and stability, while most literature reports that dementia is accompanied by poor stability, especially in the ML direction [33,52,53]. Nevertheless, since this result was not discussed in detail by the authors, we can only speculate that factors such as a different composition of the sample (i.e., presence/different proportion of individuals with Alzheimer’s disease or vascular cognitive impairment and a different woman/man ratio), as well as environmental and socio-economic backgrounds of the countries in which the studies were performed, might partly explain such a discrepancy. Generally speaking, the reduction of smoothness of gait can be attributed to alterations in limb dynamics and overall function, which can be present even in the early stages of cognitive impairment [26,54], as well as in trunk stability, especially in the presence of brain structural changes such as severe white matter lesions [34]. Moreover, individuals at increased risk of falls, such as those with cognitive impairment [55,56], have difficulties in controlling the rhythmic displacements of the trunk during gait [45], which is thus another factor able to worsen the overall smoothness of gait. 

Interestingly, the most relevant changes in HR are evident already from the early stages of cognitive decline, which is the case of ECD, while further worsening appears not to be accompanied by a corresponding deterioration in gait smoothness. This suggests that the impact of cognitive decline on gait performance is already relevant during its early or even prodromal stages, a fact that is consistent with previous observations that pointed out how the deterioration of walking abilities precedes cognitive decline and the presence of dementia [57].

### 4.3. Correlation between Cognitive Status and Gait Parameters

The results of the correlation analysis between cognitive status and spatio-temporal parameters of gait confirm its relevant role in mobility performance [58,59]. In particular, the significant moderate correlations found between cognitive scores and gait speed (0.43 for ACE-R and 0.45 for MMSE) and stride length (0.42 for ACE-R and 0.45 for MMSE) are consistent with the findings of previous studies which reported coefficient values from 0.36 to 0.60 for gait speed (vs. ACE-R [60]; vs. MMSE [17]) and 0.59 for stride length (vs. MMSE, [17]). 

There is instead a scarcity of data regarding the relationship between HR and cognitive measures, even though a number of studies have investigated the alterations of trunk accelerations in cognitively impaired people using a variety of metrics, including some quite similar in principle to HR [33,34,35,36,37,38,39,40,41,42,43,44,45,46,47,48,49,50,51,52,53,54,55,56,57], concluding that gait outcomes related to speed, regularity, predictability, and stability of trunk accelerations may suitably integrate other physical, cognitive, and behavioral measures, to better identify the extent of a cognitive impairment in the elderly. To the best of our knowledge, only Ijmker and Lamoth [35] attempted to investigate the existence of a possible relationship between HRs and MMSE score. They found a moderate positive correlation between HR AP and MMSE, similar to the observations of the present study, although slightly larger in magnitude (rho = 0.48 vs. 0.32). In contrast, Ijmker and Lamoth [35] found no significant correlation for the ML direction and did not consider the V direction. Possible reasons for the discrepancies with our findings are: (1) the fact that they did not consider the effect of gait speed, which may have some effect on HR values, as demonstrated by Lowry et al. [46]; (2) the different number of participants, which was less than a half with respect to our sample; (3) the unbalanced composition of the groups, which were predominantly composed of men.

Overall, our data suggest that gait parameters (both spatio-temporal and smoothness) are similarly influenced by the cognitive status, regardless of the way in which it is assessed, since the coefficients of correlation did not differ greatly. This would imply that while ACE-R, given its superior sensitivity, may be beneficial in better identifying the presence of dementia with respect to the MMSE, the latter appears to have sufficient capabilities for detecting the cognitive impairments associated with alterations in mobility. 

What are the clinical implications of the findings obtained in the present study? Previous research demonstrated that HR is a metric more sensitive to subtle alterations in locomotor mechanisms, with respect to spatio-temporal parameters like speed or stride length [25]. Some examples of this phenomenon were observed in individuals in the early stages of Parkinson’s disease [26] and multiple sclerosis [29]. In aging, recent research demonstrated that reductions in gait speed predicts incident dementia and cognitive decline [60], thus it is likely that the regular monitoring of trunk accelerations would probably allow the detection of changes in HR that are likely to occur earlier, with respect to those of walking speed. If such hypothesis would be confirmed by further longitudinal studies, the information provided by HR would support clinicians in the diagnosis of suspected cognitive impairment, allowing the planning of timely interventions.

### 4.4. Possible Issues Associated with the Use of IMU to Assess Gait Parameters and HR

As previously mentioned, IMU is a very appealing tool to perform the quick and inexpensive assessment of gait in a clinical setting, especially to test people with cognitive impairment, because, unlike more sophisticated equipment like optoelectronic motion capture system (which represents the gold-standard for the quantitative analysis of human movement), the test does not require a specific preparation of the individual for marker positioning and can be performed having him/her fully dressed. However, it must be noted that the validity and reliability of gait data obtained by IMU are influenced by several factors which should be considered. At first, the estimation of gait parameters could be affected by changes in sensor orientation, which may change during walking. Therefore, vertical acceleration may exhibit components in the remaining two axes which alter their actual value. 

Specific issues are also associated with the calculation of the HR, which in some cases has been criticized for poor reliability, which is not associated with the methodology by itself, but rather with a poor standardization of the measurement protocols [43]. In particular, the approach proposed by Menz et al. [31] used in the present study (which is probably the most widespread) considers the first 20 harmonics of the accelerometric signal in the frequency domain. However, as pointed out by Bellanca et al. [25], such value is justified and adequate for “regular” cadences (i.e., approximately in the range 80–135 steps/min), because very slow walking may cut a significant part of the power spectrum, thus altering the HR value. Although, in our sample, all participants satisfied this criterion, in studies involving older adults with more severe cognitive decline, who also usually exhibit significantly reduced gait speed, such an aspect should be carefully considered.

### 4.5. Limitations of the Study

Some limitations of the study are to be acknowledged, beside the technical issues previously mentioned. Although it significantly extends the amount of available data, in terms of participants tested, the number of HRs considered, and the neuropsychologic tools used to explore the relationship between gait and cognitive status, some important factors have not been included in our analysis. Firstly, we did not consider education, wealth and occupational status, which are all known to have some influence on mobility performance [61,62,63]. Thus, the generalization of the results presented here considering different socio-economic contexts should be performed cautiously. Secondly, since a non-negligible percentage of the participants were overweight or obese (31% and 13% respectively), such conditions may have introduced alterations in gait parameters, especially for their HR values [64].

## 5. Conclusions

In the present study, we have attempted to clarify the relationship between smoothness of gait and cognitive performance in a cohort of the Italian elderly, using trunk acceleration-based data acquired in a clinical setting by means of a wearable inertial sensor. The results confirm the existence of gait pattern alterations in terms of slower speed and shorter stride length, as well as a decrease of HR in all the directions investigated, which were already evident in individuals with ECD. Instead, no further worsening of smoothness of gait was detected in the presence of a more severe cognitive impairment. All the aforementioned alterations were found to be moderately correlated with the extent of the cognitive impairment in a similar way, regardless of the use of different neuropsychologic screening tools such as MMSE and ACE-R. 

Based on these findings, it is possible to state that the smoothness of gait parameters may represent a metric potentially useful in detecting subtle changes in gait possibly present in prodromal stages of dementia, but not evident from the analysis of spatio-temporal parameters alone. Such data might support the clinician in performing a more accurate diagnosis of cognitive impairment as well, in verifying the effectiveness of all those interventions targeted to overcome any possible mobility limitations in cognitively impaired individuals.

## Figures and Tables

**Table 1:** Anthropometric and clinical features of participants. Values are expressed as mean ± SD.

	Healthy Controls (HC)	Early Cognitive Decline (ECD)	Advanced Cognitive Decline (ACD)
**Participants # (F, M)**	34 (22 F, 12 M)	37 (22 F, 15 M)	19 (12 F, 7 M)
**Female/Male Ratio**	F 65%, M 35%	F 60%, M 40%	F 63%, M 37%
**Age (years)**	79.1 ± 3.9	78.8 ± 5.8	78.9 ± 4.6
**Body Mass (kg)**	64.1 ± 13.5	62.5 ± 12.9	62.6 ± 17.1
**Height (cm)**	159.9 ± 8.6	159.3 ± 8.8	158.1 ± 9.7
**Mini Mental State Examination (MMSE)**	27.6 ± 1.7	22.0 ± 1.5	11.8 ± 5.1
**Addenbrooke’s Cognitive Examination Revised (ACE-R)**	77.8 ± 11.1	55.5 ± 9.7	25.0 ± 15.4

**Table 2:** Spatio-temporal and smoothness-of-gait parameters calculated for the three groups of elderly. Values are expressed as mean ± SD.

Gait Parameter	Healthy Controls (HC)	Early Cognitive Decline (ECD)	Advanced Cognitive Decline (ACD)
**Gait speed (m s^−1^)**	0.92 ± 0.23	0.68 ± 0.30 ^a^	0.63 ± 0.25 ^a^
**Stride length (m)**	1.03 ± 0.23	0.81 ± 0.32 ^a^	0.73 ± 0.23 ^a^
**Cadence (steps min^−1^)**	107.3 ± 8.5	100.6 ± 12.6	101.7 ± 11.8
**Stance phase (% GC)**	61.3 ± 2.0	61.9 ± 2.3	62.0 ± 1.6
**Swing phase (% GC)**	38.8 ± 1.9	38.1 ± 2.3	37.4 ± 2.8
**Double support phase (% GC)**	22.3 ± 2.0	23.8 ± 2.3	24.0 ± 1.7
Harmonic ratio (HR) anteroposterior (AP) direction *	3.37 ± 0.69	2.49 ± 0.88 ^a^	2.31 ± 0.76 ^a^
HR mediolateral (ML) direction *	2.40 ± 0.72	2.03 ± 0.53	2.05 ± 0.55
HR vertical (V) direction *	3.70 ± 0.93	2.60 ± 0.87 ^a^	2.60 ± 0.88 ^a^

^a^ significant difference vs. HC after Bonferroni correction; * controlled for gait speed; GC: Gait Cycle.

**Table 3:** Spearman’s coefficients for correlations between spatial-temporal and smoothness of gait parameters and scores obtained from the neuropsychological assessment.

Gait Variables		MMSE	ACE-R
**Spatial-temporal parameters**	**Gait speed**	0.449 ^††^	0.430 ^††^
	**Stride length**	0.446 ^††^	0.422 ^††^
	**Cadence**	0.199	0.191
	**Stance phase**	−0.156	−0.143
	**Swing phase**	0.192	0.182
	**Double support phase**	−0.153	−0.149
**Harmonic Ratio**	HR AP direction *	0.323 ^††^	0.303 ^††^
	HR ML direction *	0.213 ^†^	0.251^†^
	HR V direction *	0.259 ^†^	0.207 ^†^

^†^*p* < 0.05; ^††^ *p* < 0.01; * controlled for gait speed; ACE-R: Addenbrooke’s Cognitive Examination (Revised); MMSE: Mini Mental State Examination; AP: antero-posterior; ML: medio-lateral; V: vertical.
